# A comprehensive comparison between cementless and cemented fixation in the total knee arthroplasty: an updated systematic review and meta-analysis

**DOI:** 10.1186/s13018-021-02299-4

**Published:** 2021-03-05

**Authors:** Yuan Liu, Yi Zeng, Yuangang Wu, Mingyang Li, Huiqi Xie, Bin Shen

**Affiliations:** 1 Department of Orthopaedic Surgery, West China Hospital, West China Medical School, Sichuan University, 37# Guoxue Road, Chengdu, 610041 People’s Republic of China; 2 Department of Orthopaedic surgery, National Clinical Research Center for Geriatrics, West China Hospital, Sichuan University, 37# Guoxue Road, Chengdu, 610041 People’s Republic of China

**Keywords:** Total knee arthroplasty, Cement, Cementless, Systematical review, Meta-analysis

## Abstract

**Background:**

Whether the cement should be used in the total knee arthroplasty (TKA) was still in controversy. This meta-analysis was performed to compare the efficacy of two kinds of fixation.

**Methods:**

Randomized controlled trials (RCTs), prospective/retrospective observational studies from PubMed (on 2019 September), EMBASE (on 2019 September), and the Cochrane Central Register of Controlled Trials (CENTRAL) and Web of Science (on 2019 September) were searched. Only studies followed more than 2 years was included for the review. The PRISMA guidelines and Cochrane Handbook were adopted to assess the quality of the results reported in included studies to ensure that the results of our meta-analysis were reliable and veritable. The continuous and dichotomous outcomes were collected in a standard form, and the data were analyzed by Review Manager 5.3 software. Finally, the results were presented in the Forest plots.

**Results:**

Twenty-six studies involving 2369 patients in cementless TKA and 2654 patients in cemented TKA were included. The rate of revision was not significantly different in two groups (*p* = 0.55). More than eight reasons caused revision were found in our study, the aseptic loosing was the most common, followed by the periprosthetic joint infection (PJI), neither was significantly different (*p* = 0.88 and 0.45, respectively). While significantly better long-term functional recovery was found in cementless TKA in terms of Knee Society Function Score (*p* = 0.004) and manipulation under anesthesia (*p* = 0.007).

**Conclusion:**

Cementless fixation did not decrease the rate of revision after the total knee arthroplasty compared with the cemented fixation, while the long-term functional recovery was significantly better in the cementless group.

**Supplementary Information:**

The online version contains supplementary material available at 10.1186/s13018-021-02299-4.

## Background

As the gold standard of fixation method in total knee arthroplasty (TKA), cemented fixation occupied 93.5% in 2010 [1]. There were a series of advantages in conventional cemented fixation in TKA. Firstly, the cemented fixation allows for small bone cut defects, which required less technical challenge compared with the uncemented fixation [2]. Secondly, the cemented fixation could deliver antibiotics into the joint to prevent infection [3]. Thirdly, as an effective barrier, cement could insulate the polyethylene debris from the articular surface and prevent the osteolysis [4]. Therefore, because of the abundant clinical experience and great clinical results, cemented fixation was still most used in TKA. An analysis using New Zealand Joint Registry (NZJR) data revealed that most (91.5%) of primary TKA were fully cemented with 4.8% hybrid and 3.7% uncemented in 96,519 primary TKAs from 1998 to 2017 [5].

However, accompanied with the increasing demand of TKA, the patients underwent TKA are becoming younger and younger [6]. It was predicted that more than half of patients underwent TKA was contributed by patients younger than 65 years old by 2030 [6]. This posed a difficulty to the development of TKA, for the more active lifestyle was needed by younger patients. Therefore, the concern of bone resorption in the bone-cement interface would make the dominance of cemented fixation challenging [7]. Although the preliminary results of cementless fixation was proved discouraging, cementless TKA in young patients was found to have comparable midterm results to cemented TKA [8]. With a biologic bone-implant interface, cementless fixation was determined to prevent the osteolysis and decrease the risk of aseptic loosening, especially in young patients. In addition, with the development of prosthesis design and material renovation, cementless TKA has been introduced to extend the life of prosthesis [9].

Therefore, this study was performed to compare the rate of revision, reasons of revision, functional recovery, and rate of complications in two kinds of fixation in TKA. We hypothesized that the cementless fixation was not inferior to the cemented fixation in terms of rate of revision and functional recovery.

## Methods

The guidelines listed in the Preferred Reporting Items for Systematic Reviews and Meta-Analyses (PRISMA) was the basis of this systematic review and meta-analysis (The PRISMA checklist was shown in the Supplementary Material) [10].

### Search strategy

MEDLINE (1950 to date), PubMed (1966 to date), EMBASE (1974 to date), the Cochrane Central Register of Controlled Trials, the Wanfang database (1982 to date), and the Web of Science were systematically searched for studies on cementless fixation in total knee arthroplasty on 30 August 2019. “Knee, knee replacement, knee arthroplasty, total knee replacement, TKR, total knee arthroplasty, TKA,” and “cementless, cemented, uncemented” were used as key words in connection with AND or OR. Meta-analyses were identified by the third reviewer. Then, the references of these meta-analyses were screened to find additional relevant studies. Another reviewer tried to contact expert informants by email to search for unpublished studies. Finally, two reviewers independently assessed the studies, and any discrepancies were resolved by a discussion with the other reviewers.

### Inclusion and exclusion criteria

Studies were included according to the PICOS criteria: (1) population: patients experiencing TKA who were demographically alike; (2) intervention and control: cementless and cemented fixation in TKA; (3) outcomes: patients followed at least 2 years, and rate of revision, reasons of revision, functional recovery, and rate of complication were reported; (4) study design: randomized controlled trial (RCT), prospective, or retrospective observational studies.

Studies were excluded if (1) relevant outcomes were missing or (2) the quality assessment was low (RCT < 5, non-RCTs < 20) [11, 12]. (3) The groups in study were not fully cementless and fully cemented that the hybrid fixation was not included in this study.

### Quality assessment

A modified seven-point JADAD scale was adopted to assess the methodological quality of the RCTs [11]. The scale considers five items, namely, randomization, concealment of allocation, double blinding, withdrawals, and dropouts [11]. Based on the Cochrane Handbook, two reviewers independently evaluated the quality of the included RCTs. There was no disagreement between the two reviewers’ ratings.

Two reviewers evaluated the quality of non-RCTs by Methodological Index for Non-Randomized Studies scale (MINORS), which has a range of scores from 0 to 24 [12]. Unified consensus was obtained if there were any different opinions.

### Data extraction

The relevant data, including the authors, year of publication, country, baseline information of participants, prosthesis design, revision rate, power analysis, and the length of follow-up were independently extracted by two reviewers using a standard data extraction form.

To compare the two kinds of fixation in TKA, the outcomes were summarized in three parts. The first part was the rate of revision and reasons of revision, which was the primary outcome of our study. The second part was the postoperative functional recovery, including the Knee Society (KSS) knee and function scores, Oxford knee scores, range of motion (ROM), and manipulation under anesthesia. The third part was the rate of complication, including deep vein thrombosis (DVT) and all infection (including superficial wound infection and periprosthetic joint infection).

### Statistical analysis

The Review Manager 5.3 (Nordic Cochrane Center, Copenhagen, Denmark) was used to perform the meta-analysis. The final results were shown in Forest plots. Mean differences (MD) or standard mean differences (SMD) were used to weigh the effect size for continuous outcomes, and relative risks (RR) were used for dichotomous outcomes. The *I*^2^ statistic was used to test for heterogeneity across the included studies [11]. A *p* value ≤ 0.1 or an *I*^2^ > 50% was regarded as proof of heterogeneity. A random-effects model is used to synthesize results with high heterogeneity and is more conservative than a fixed effects model. Therefore, a random-effects model was used to alleviate the effect caused by high heterogeneity, and a fixed effects model was used when statistical evidence showed low heterogeneity.

## Results

### Search results

**Fig. 1:**
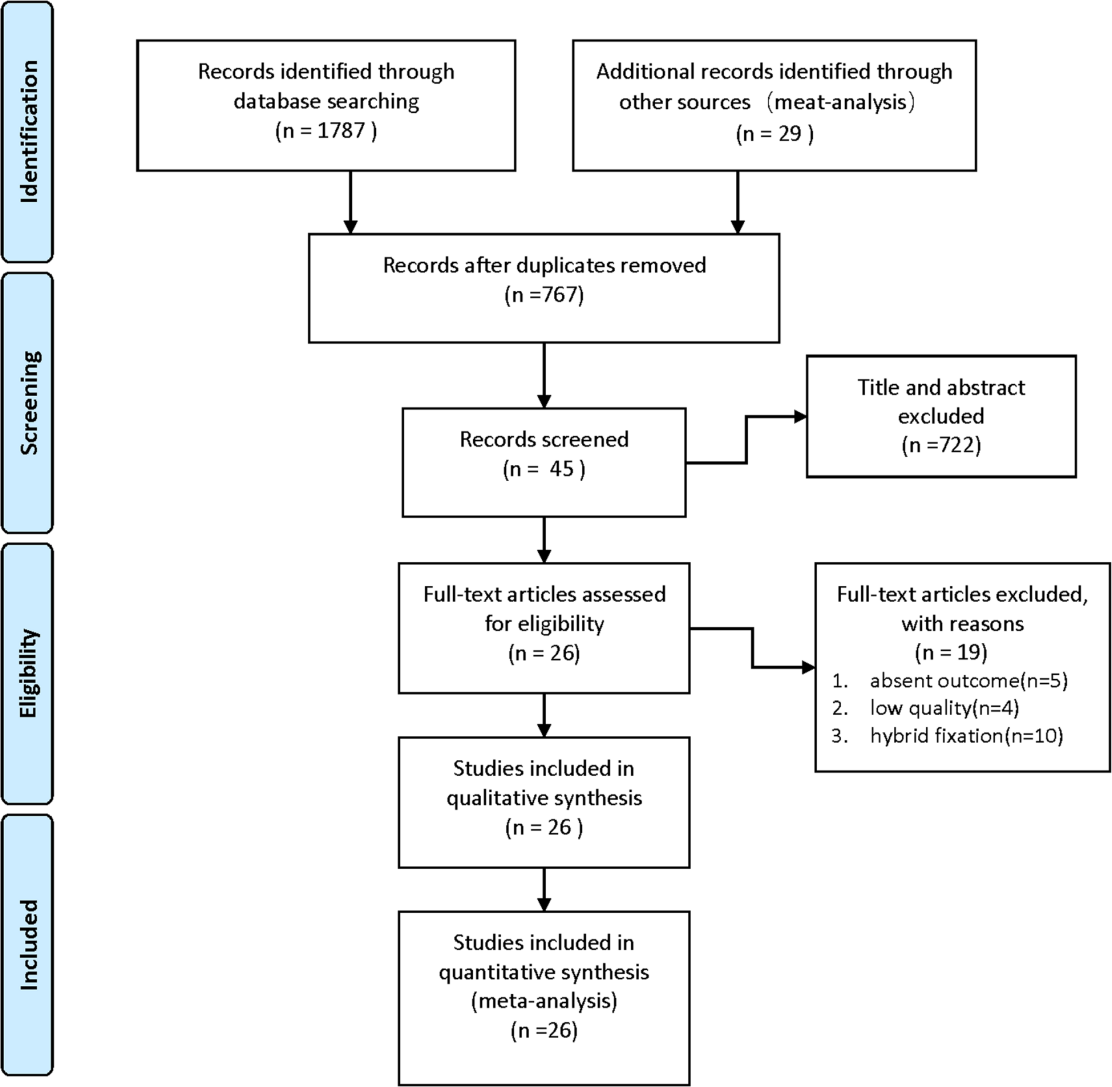
The flowchart of the study selection

As shown in Fig. 1, a total of 1787 articles were obtained from the databases via the search strategy. After removing duplicates, 767 articles were screened. From among them, 722 articles were removed after reading the title and abstract based on the inclusion criteria. Then, 19 studies were excluded on the basis of exclusion criteria. Finally, 11 RCTs [2, 13–22] and 15 non-RCTs [23–37] were included in this study.

### Baseline information and quality assessment

Twenty-six studies [2, 13–37] involving 2369 patients in cementless group and 2654 patients in cemented group were included in this review and meta-analysis. The baseline information including study design, demographical data, revision rate, prosthesis, and length of follow-up were clearly listed in Table 1. Especially, the duration of follow-up was same for cemented and cementless groups. And the length of the follow-up in all included studies ranged from 2 years to 16.6 years.

**Table 1:** The baseline information of studies compared cementless with cement fixation in TKA

			Cementless/cement							
Studies	country	Study design	Cases	Age	BMI	Female	Revision rate(%)	Prosthesis	Power analysis	Follow-up
**Abu-rajab 2006** [23]	UK	Non-RCT	20/18	69/71	–	11/8	–	–	Y	2 years
**Anis 2019** [24]	USA	Non-RCT	133/132	60/62	33/33	51/44	2.3/1.5	–	N	2 years
**Bagsby 2016** [25]	USA	Non-RCT	145/154	62.7/58.8	44.7/45.6	102/122	0.7/13	Stryker Triathlon	N	3.65 years
**Baker 2007** [13]	UK	RCT	224/277	71/70		103/121	8.9/8.7	PFC	N	8.7 years
**Boyle et al. 2017 **[26]	USA	Non-RCT	154/171	59.6/64.9	37.4/37.4	97/128	3.9/3.5	Stryker Triathlon	N	5.7 years
**Carlsson 2005** [14]	Sweden	RCT	27/29	74/72	–	20/22	3.7/0	PFC	N	5 years
**Dodd 1990** [27]	UK	Non-RCT	18/18	–	–	15/15	5.6/5.6	PCA	N	5 years
**Duffy 1998** [28]	USA	Non-RCT	55/51	54/65	29.07/27.63	23/24	16.4/5.9	PFC	N	10.2 years
**Fernandez-Fairen 2013** [15]	Switzerland	RCT	74/71	61/60	29.1/30.5	55/54	0/1.6	NexGen CR	Y	5 years
**Fricka 2015** [16]	USA	RCT	47/46	60.2/58.6	31.4/32.7	29/33	2.1/2.2	NexGen CR	Y	2 years
**Fricka 2019** [17]	USA	RCT	41/44	59.8/58.4	31.4/31.9	26/31	4.9/2.3	NexGen CR	Y	5 years
**Gao 2009** [18]	Sweden	RCT	19/22	–	–	–	5.3/0	NexGen CR	Y	2 years
**Kamath 2011** [29]	USA	Non-RCT	100/312	55/63	–	–	2/1.6	NexGen CR	N	5 years
**Karachalios 2018** [30]	Greek	Non-RCT	54/54	63.2/63.8	32/31.5	36/37	–	aMP system	Y	8.6 years
**Khaw 2002** [19]	UK	RCT	177/219	71/70	–	103/121	3.95/4.11	PFC	N	7.3 years
**Kim 2014** [2]	Korea	RCT	80/80	54.3/54.3	27.8/27.8	63/63	1.25/0	NexGen CR	Y	16.6 years
**McCaskie 1998** [20]	UK	RCT	58/81	70.2/68.8	–	32/49	–	PFC	N	5 years
**Miller et al. 2017 **[31]	USA	Non-RCT	200/200	64.3/64.4	33.9/33.1	125/125	3.5/4	Stryker Triathlon	N	5.3 years
**Nam 2019** [21]	USA	RCT	76/65	61.3/63	31.1/31.3	36/34	0/1.5	Stryker Triathlon	Y	2 years
**Pap 2018** [32]	Hungary	Non-RCT	134/140	59/69		53/64	1.5/1.4	SanatSwing	N	2 years
**Park 2011** [22]	Korea	RCT	50/50	58.4/58.4	26.6/26.6	39/39	2/0	NexGen CR	Y	13.6 years
**Pecina 2000** [33]	Croatia	Non-RCT	87/44	57/62	–	–	22.99/15.91	PCA	N	7.3 years
**Prudhon 2017** [34]	France	Non-RCT	100/100	72.3/73.2	–	57/59	5/10	NEW WAVETM	N	12.1 years
**Rand 1991** [35]	USA	Non-RCT	59/59	57/66	29.4/24.4	24/25	–	PFC	N	2.8 years
**Rosenberg 1990** [36]	USA	Non-RCT	132/139	59/70		77/82	4.5/5.8	–	N	3.6 years
**Sinicrope et al. 2018 **[37]	USA	Non-RCT	108/85	62/60	45.6/45	82/67	4.63/25.88	Stryker Triathlon	Y	5 years

*RCT* randomized controlled trial, *BMI* body mass index, *PFC* press-fit condylar, *PCA* porous-coated anatomic, *CR* cruciate-retaining

The JADAD score of 11 RCTs were listed in Table 2, both of them were ≧ 5, four of them [2, 15, 21, 22] got 7 points. The MINORS scores of 15 non-RCTs were listed in Table 3, both of them were ≧ 20, only 1 of them [37] got 24 points.

**Table 2:** The quality assessment of RCTs

Studies	Randomization	Concealment of allocation	Double blinding	Withdrawal and dropout	Total score
**Baker 2007** [13]	1	2	2	1	6
**Carlsson 2005** [14]	2	2	1	1	6
**Fernandez-Fairen 2013** [15]	2	2	2	1	7
**Fricka 2015** [16]	2	2	1	1	6
**Fricka 2019** [17]	2	2	1	1	6
**Gao 2009** [18]	2	2	1	1	6
**Khaw 2002** [19]	1	2	2	1	6
**Kim 2014** [2]	2	2	2	1	7
**McCaskie 1998** [20]	1	2	1	1	5
**Nam 2019** [21]	2	2	2	1	7
**Park 2011** [22]	2	2	2	1	7

**Table 3:** The quality assessment of non-RCTs

studies	A clearly stated aim	Inclusion of consecutive patients	Prospective data collection	Endpoints appropriate to the aim of the study	Unbiased assessment of the study endpoint	A follow-up period appropriate to the aims of study	Less than 5% loss to follow-up	Prospective calculation of the sample size	An adequate control group	Contemporary groups	Baseline equivalence of groups	Adequate statistical analyses	Total score
**Abu-rajab 2006** [23]	2	2	2	2	2	2	0	2	2	2	2	2	22
**Anis 2019** [24]	2	2	0	2	2	2	2	0	2	2	2	2	20
**Bagsby 2016** [25]	2	2	2	2	2	2	2	0	2	1	2	2	21
**Boyle 2017**	2	2	2	2	2	2	0	0	2	2	2	2	20
**Dodd 1990** [27]	2	2	2	2	2	2	2	0	2	2	2	2	22
**Duffy 1998** [28]	2	2	2	2	2	2	0	0	2	2	2	2	20
**Kamath 2011** [29]	2	2	2	2	2	2	1	0	2	2	2	2	21
**Karachalios 2018** [30]	2	2	2	2	2	2	2	2	2	0	2	2	22
**Miller 2017**	2	2	2	2	2	2	2	0	2	2	2	2	22
**Pap 2018** [32]	2	2	2	2	2	2	2	0	2	2	2	2	22
**Pecina 2000** [33]	2	2	2	2	2	2	0	0	2	2	2	2	20
**Prudhon 201 7**[34]	2	2	2	2	2	2	2	0	2	0	2	2	20
**Rand 1991** [35]	2	2	2	2	2	2	2	0	2	2	2	2	22
**Rosenberg 1990** [36]	2	2	2	2	2	2	2	0	2	2	2	2	22
**Sinicrope 2018**	2	2	2	2	2	2	2	2	2	2	2	2	24

### Rate of revision and reasons of revision

**Fig. 2:**
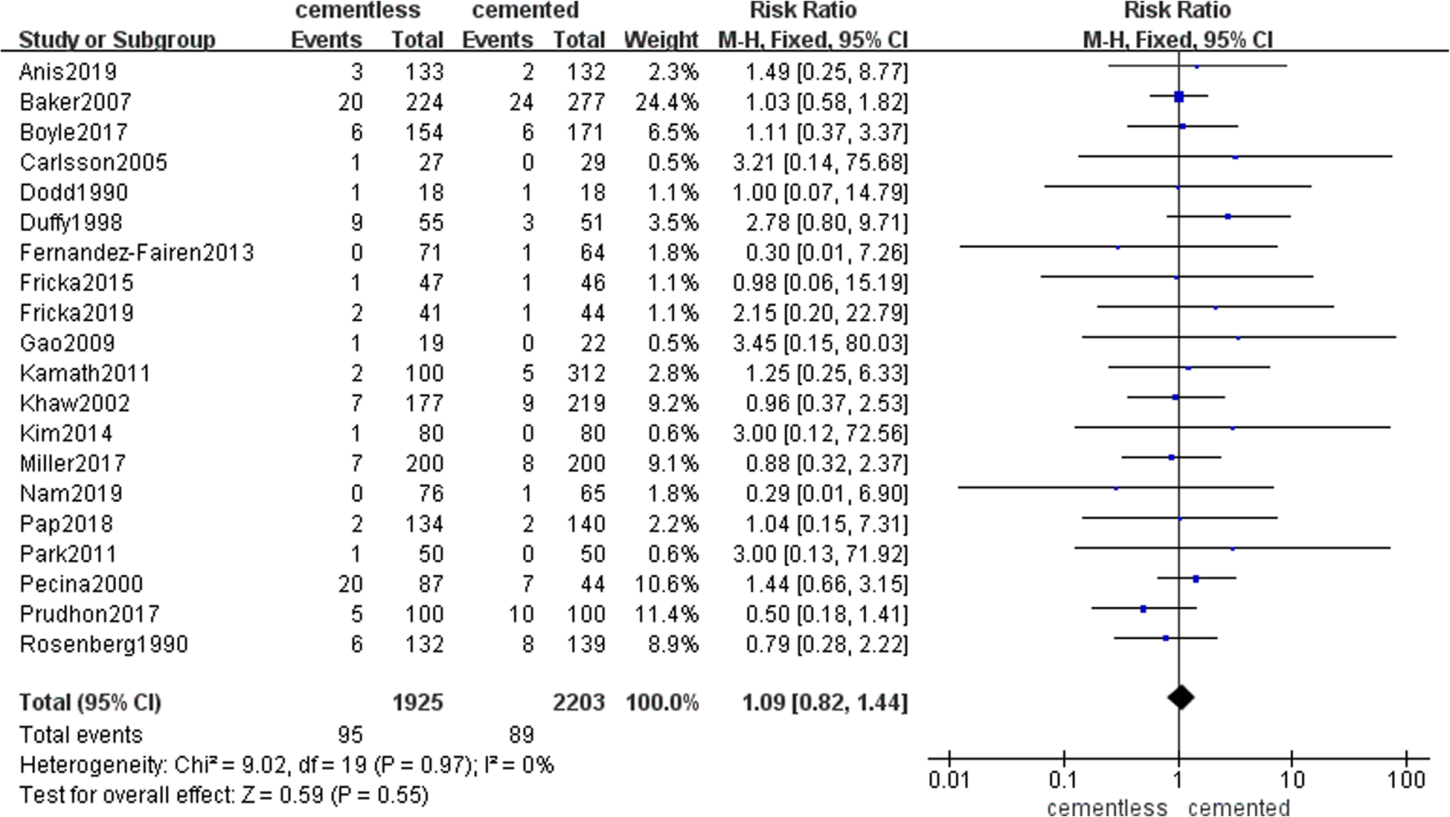
The frost blot about the rate of revision

A total of 20 studies involving 1925 patients in cementless group and 2203 patients in cemented group reported the rate of revision during the follow-up. There were 95(4.9%) patients in the cementless group and 89 (4%) patients in the cemented group underwent the revision for all kinds of reasons. Pooled results shown that there was no significant difference between the rate of revision during the at least 2 years of follow-up in two groups (RR = 1.09, 95% CI [0.82, 1.44], *p* = 0.55; Fig. 2).

**Fig. 3:**
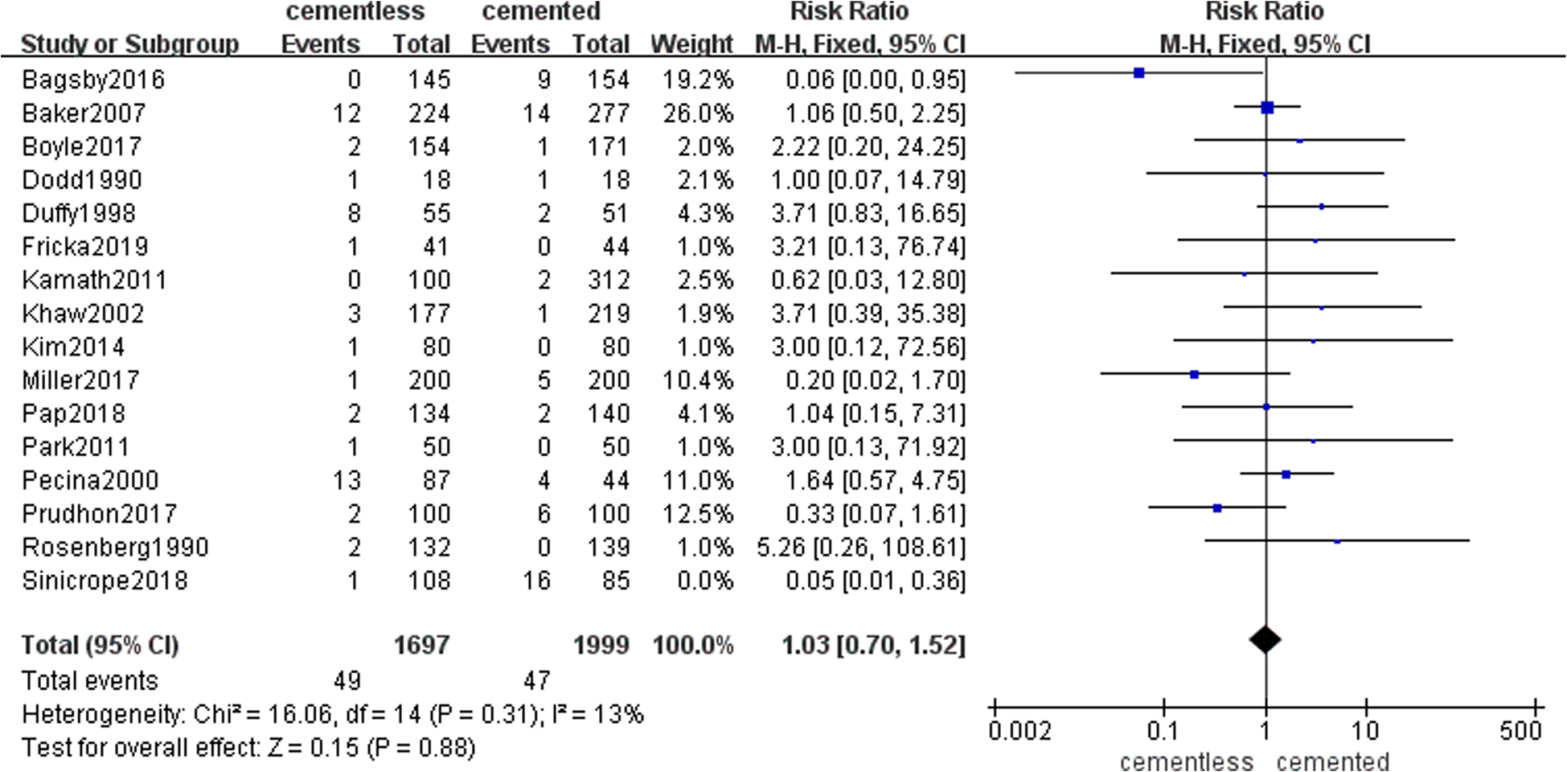
The frost blot about the rate of aseptic loosing

**Fig. 4:**
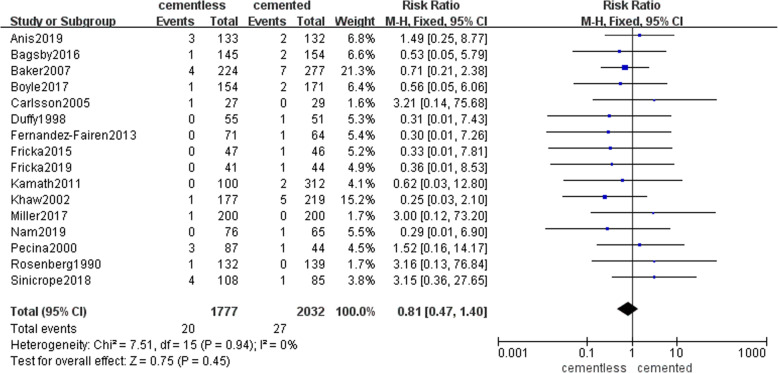
The frost blot about the rate of periprosthetic joint infection

Among reasons that caused revision, aseptic loosing was the most common, next was the periprosthetic joint infection (PJI). Fifteen studies with 1697 patients in the cementless group and 1999 patients in the cemented group recorded the rate of aseptic loosing caused the revision (49 (2.9%) and 47(2.4%), respectively). Pooled result presented that there was no significant difference in two groups (RR = 1.03, 95% CI [0.7, 1.52], *P* = 0.88; Fig. 3). Sixteen studies with 1777 patients in the cementless group and 2032 patients in the cemented group reported the rate of PJI caused the revision (20 (1.1%) and 27(1.3%), respectively). Pooled result presented that there was no significant difference in two groups (RR = 0.81, 95% CI [0.47, 1.4], *p* = 0.45; Fig. 4). The specific number of revisions and other reasons were listed in Table 4 in detail.

**Table 4:** The specific rate of revision caused by all kinds of reasons

	Cementless/cemented (number (%))										
Studies	Patients	Number of revisions	Aseptic loosing	PJI	Instability	Polyethylene wear	Exchange of tibial polyethylene insert	Periprosthetic fracture	Patella dislocation	Stiffness	Other reasons
**Anis 2019** [24]	133/132	3(2.3)/2(1.5)		3(2.3)/2(1.5)							
**Baker 2007** [13]	224/277	20(8.9)/24(8.7)	12(5.4)/14(5)	4(1.8)/7(2.5)	2(0.9)/0(0)	0(0)/1(0.4)	2(0.9)/2(0.7)				
**Boyle 2017**	154/171	6(3.9)/6(3.5)	2(1.3)/1(0.6)	1(0.6)/2(1.2)							3(1.9)/3(1.8)
**Carlsson 2005** [14]	27/29	1(3.7)/0(0)		1(3.7)/0(0)							
**Dodd 1990** [27]	18/18	1(5.6)/1(5.6)	1(5.6)/1(5.6)								
**Duffy 1998** [28]	55/51	9(16.4)/3(5.9)	8(14.5)/2(3.9)	0(0)/1(2)							1(1.8)/0(0)
**Fernandez-Fairen 2013** [15]	71/64	0(0)/1(1.6)		0(0)/1(1.6)							
**Fricka 2015** [16]	47/46	1(2.1)/1(2.2)		0(0)/1(2.2)	1(2.1)/0(0)						
**Fricka 2019** [17]	41/44	2(4.9)/1(2.3)	1(2.4)/0(0)	0(0)/1(2.3)				1(2.4)/0(0)			
**Gao 2009** [18]	19/22	1(5.3)/0(0)									1(5.3)/0(0)
**Kamath 2011** [29]	100/312	2(2)/5(1.6)	0(0)/2(0.6)	0(0)/2(0.6)	1(1)/0(0)						1(1)/1(0.3)
**Khaw 2002** [19]	177/219	7(3.95)/9(4.11)	3(1.69)/1(0.46)	1(0.56)/5(2.28)		0(0)/1(0.46)	3(1.69)/2(0.91)				
**Kim 2014** [2]	80/80	1(1.25)/0(0)	1(1.25)/0(0)	-		-	-				
**Miller 2017**	200/200	7(3.5)/8(4)	1(0.5)/5(2.5)	1(0.5)/0(0)	2(1)/2(1)				1(0.5)/0(0)		2(1)/1(1)
**Nam 2019** [21]	76/65	0(0)/1(1.5)		0(0)/1(1.5)							
**Pap 2018** [32]	134/140	2(1.5)/2(1.4)	2(1.5)/2(1.4)								
**Park 2011** [22]	50/50	1(2)/0(0)	1(2)/0(0)								
**Pecina 2000** [33]	87/44	20(22.99)/7(15.91)	13(14.94)/4(9.09)	3(3.45)/1(2.27)			0(0)/2(4.55)	1(1.15)/0(0)	3(3.45)/0(0)		
**Prudhon 2017** [34]	100/100	5(5)/10(10)	2(2)/6(6)	0(0)/1(1)				3(3)/1(1)		0(0)/1(1)	0(0)/1(1)
**Rosenberg 1990** [36]	132/139	6(4.5)/8(5.8)	2(1.5)/0(0)	1(0.8)/0(0)	0(0)/2(1.4)						3(2.3)/6(4.3)
***Total***	***1925/2203***	***95(4.9)/89(4)***	***49(2.5)/38(1.7)***	***15(0.8)/25(1.1)***	***6(0.3)/4(0.2)***	***0(0)/2(0.09)***	***5(0.2)/6(0.3)***	***5(0.3)/1(0.05)***	***4(0.2)/0(0)***	***0(0)/1(0.05)***	***11(0.57)/12(0.54)***
***Rate of revision***	***4108***	***184(4.5)***	***87(2.1)***	***40(0.97)***	***10(0.24)***	***2(0.05)***	***11(0.24)***	***6(0.14)***	***4(0.1)***	***1(0.02)***	***23(0.56)***

*PJI* periprosthetic joint infection

### Functional recovery

**Fig. 5:**
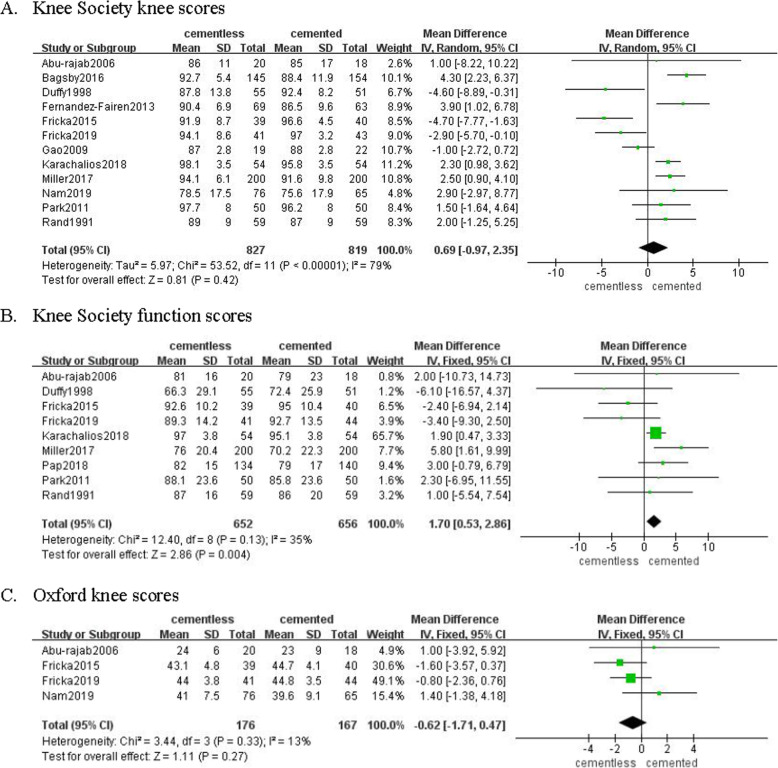
The frost blot about the functional recovery

Twelve studies involving 827 patients in cementless group and 819 patients in cemented group recorded the Knee Society knee score. Pooled results revealed there was no significant difference regarding Knee Society knee score between two groups (MD = 0.69, 95% CI [− 0.97, 2.35], *p* = 0.42; Fig. 5a). Nine studies involving 652 patients in cementless group and 656 patients in cemented group recorded the Knee Society function score, which was significantly higher in the former group (MD = 1.70, 95% CI [0.53, 2.86], *p* = 0.004; Fig. 5b). Four studies involving 176 patients in cementless group and 167 patients in cemented group recorded the Oxford knee score. Pooled results revealed that the kind of fixation did not make a difference on Oxford knee score in short duration (MD = − 0.62, 95% CI [− 1.71, 0.47], *p* = 0.27; Fig. 5c).

**Fig. 6:**
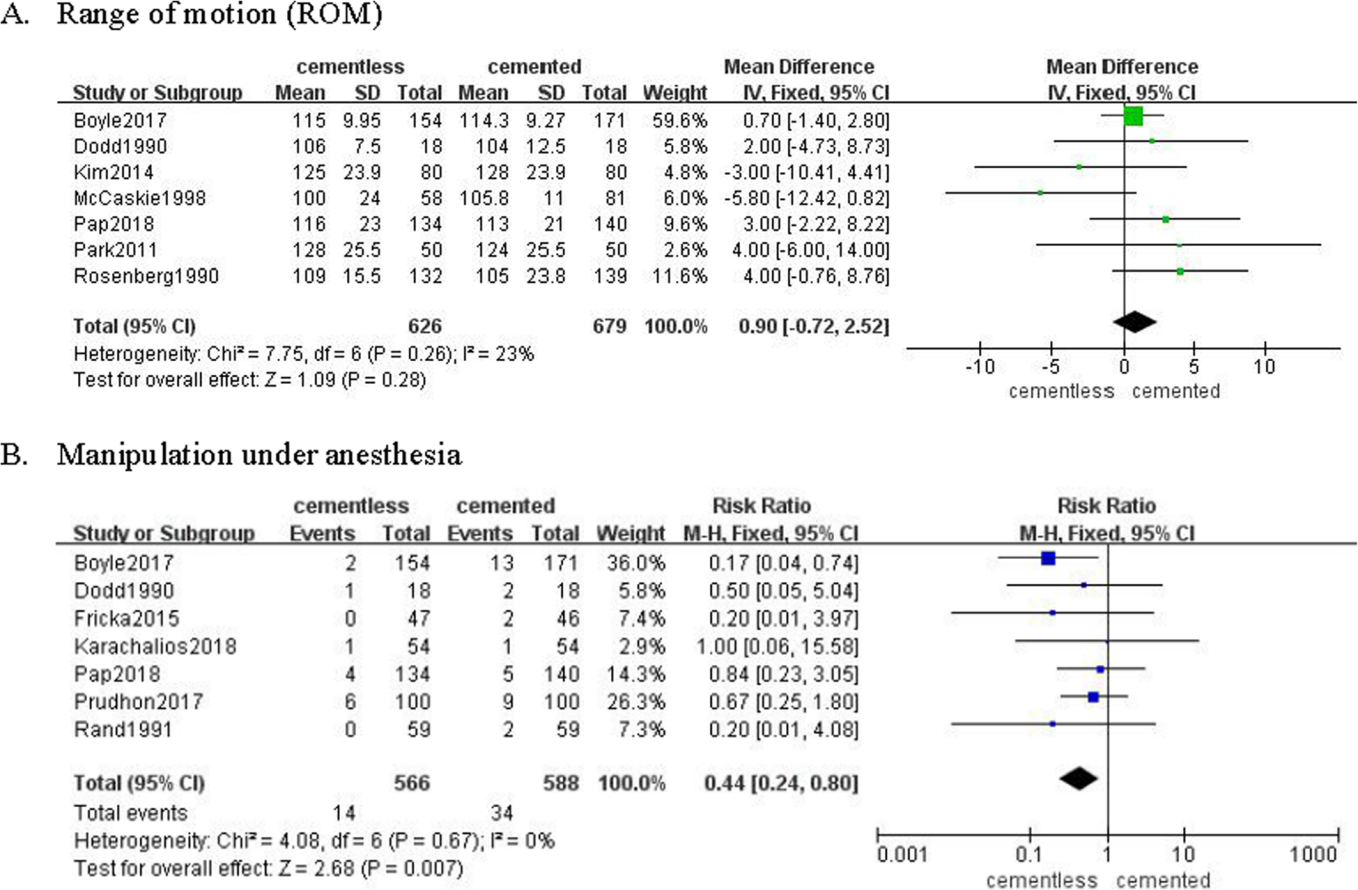
The frost blot about the knee motion

Seven studies involving 626 patients in cementless group and 679 patients in cemented group reported the range of motion (ROM) following TKA. Pooled results revealed the ROM was not significantly different in two groups (MD = 0.9, 95% CI [− 0.72, 2.52], *P* = 0.28; Fig. 6a). However, 7 studies involving 566 patients in the cementless group and 588 patients in the cemented group revealed that the rate of manipulation under anesthesia was significantly more in the cemented group (RR = 0.44, 95% CI [0.24, 0.80], *p* = 0.007; Fig. 6b).

### Complications

**Fig. 7:**
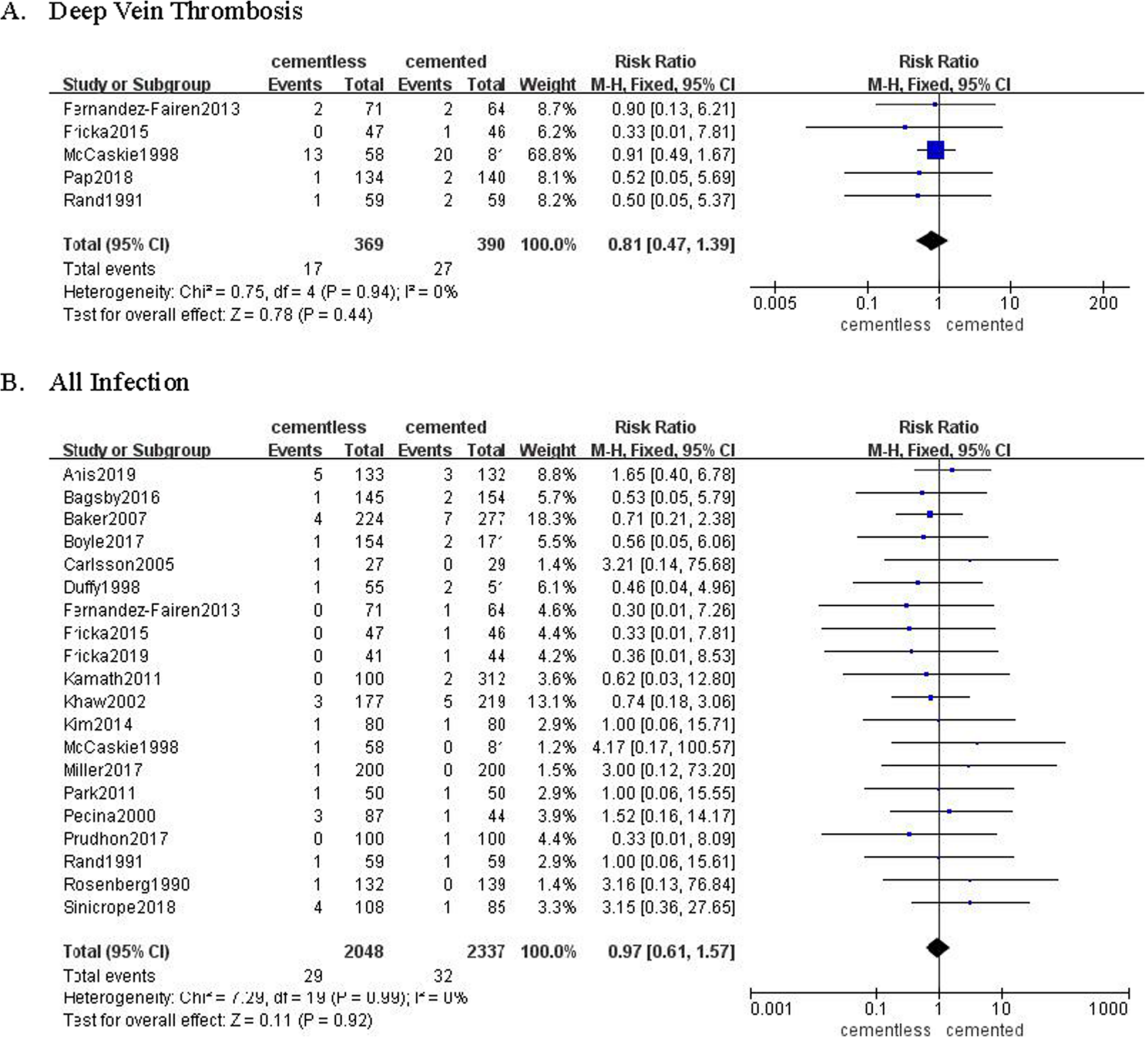
The frost blot about the rate of complications

Five studies involving 369 patients in cementless group and 390 patients in cemented group recorded the rate of deep vein thrombosis (DVT) following TKA. There were 17 (4.6%) in cementless group and 27 (6.9%) in cemented group diagnosed as DVT, while it was not significantly different (RR = 0.81, 95% CI [0.47, 1.39], *p* = 0.44; Fig. 7a). Twenty studies involving 2048 patients in the cementless group and 2337 patients in the cemented group recorded the rate of all infection following TKA. There were 29 (1.4%) patients in the cementless group and 32 (1.4%) patients in the cemented group diagnosed as superficial 3rwound infection or PJI. The pooled results shown insignificant difference (RR = 0.97, 95% CI [0.61, 1.57], *P* = 0.92; Fig. 7b).

## Discussion

Compared with the published review and meta-analysis [38–42], the most prominent advantage of our study was that a minimum 2 years length of follow-up criteria was used for screening studies and analyzing these together.

Survivorship of prosthesis was the most important endpoint in TKA [43]. Rate of revision and reasons caused revision were primary outcomes in our study. Although the pooled results shown insignificant difference, the cementless fixation presented significant superiority in studies followed more than 2 years and less than 5.5 years. In addition, although a relatively longer follow-up was needed to compare the true difference regarding the rate of revision between two kinds of fixation in TKA. It has been reported that 3 to 50% primary TKAs underwent revision within the first 5 years [44, 45]. More than 8 kinds of reasons that caused revision after TKA were found, aseptic loosing was the most common. Among all causes of revision, rate of aseptic loosing was 2.1%, followed by the periprosthetic joint infection (PJI, 0.97%), instability (0.24%), exchange of tibia polyethylene insert (0.24%), periprosthetic fracture (0.14%), patella dislocation (0.1%), polyethylene wear (0.05%), stiffness (0.02%), and other reasons (0.56%). Consistent with the rate of revision, rate of aseptic loosing was significantly decreased in the cementless fixation in studies followed more than 2 years and less than 5 years. However, other reasons including PJI were not significantly different between two groups. Therefore, it is induced that aseptic loosing was possibly easier happened in bone-cement interface.

In terms of functional recovery after TKA, patients in cementless group had better Knee Function Score compared with patients in cemented group. Although ROM was not significantly different, there were significantly less patients in cementless group required manipulation under anesthesia. A possible explanation for the better recovery in cementless TKA was that relevant complications including osteolysis, anterior knee pain was more common in the cemented group. In addition, it is worth noting that the length of follow-up in studies reported the Knee Society Function score was all more than 8.5 years, and also in the studies reported manipulation under anesthesia mostly followed more than 5 years. It could be concluded that cementless fixation might present long-term advantages regarding the functional recovery.

Along with the development of manufacture and biomaterials including highly porous metals, cross-linked polyethylene, and corrections in initial cementless designs, some recent publications show successful results in long-term follow-up of cementless fixation [46]. Interest on cementless fixation increased as more young patients underwent TKA. Moreover, cementless TKA presented lower revision rates compared with cemented fixation in morbidly obese patients [25]. A possible reason was that greater stress was placed on the bone–implant interface when patients were more active or obese [47]. Therefore, inferior performance of cemented TKA in younger and obese patients made the advent of cementless an alternative way to offer long-term fixation. What is more, a study published in 2019 reported that cementless TKA costed much less than cemented TKA [48].

There are several shortcomings in our study. Firstly, studies followed longer than 10 years was not enough. Secondly, the prosthesis design used in included studies was not totally same, which might increase bias of risk. Thirdly, there were only 11 RCTs in 26 studies included in our study decreased the level of evidence.

## Conclusion

Cementless fixation did not decrease the rate of revision after the total knee arthroplasty compared with the cemented fixation, while the long-term functional recovery was significantly better in the cementless group.

## Supplementary Information

**Additional file 1.** PRISMA 2009 Checklist.

## Data Availability

The datasets used and/or analyzed during the current study are available from the corresponding author on reasonable request.

## Abbreviations

TKA: Total knee arthroplasty
DVT: Deep vein thrombosis
ROM: Range of motion
PJI: Periprosthetic joint infection
